# Cancer Stem Cells in Melanoma: Drivers of Tumor Plasticity and Emerging Therapeutic Strategies

**DOI:** 10.3390/ijms26157419

**Published:** 2025-08-01

**Authors:** Adrian-Horațiu Sabău, Andreea-Cătălina Tinca, Raluca Niculescu, Iuliu Gabriel Cocuz, Andreea Raluca Cozac-Szöke, Bianca Andreea Lazar, Diana Maria Chiorean, Corina Eugenia Budin, Ovidiu Simion Cotoi

**Affiliations:** 1 Doctoral School of Medicine and Pharmacy, University of Medicine, Pharmacy, Sciences and Technology “George Emil Palade” of Targu Mures, 540142 Targu Mures, Romania; adrian-horatiu.sabau@umfst.ro; 2 Pathophysiology Department, University of Medicine, Pharmacy, Sciences and Technology “George Emil Palade” of Targu Mures, 540142 Targu Mures, Romania; raluca.niculescu@umfst.ro (R.N.); iuliu.cocuz@umfst.ro (I.G.C.); andreea-raluca.szoke@umfst.ro (A.R.C.-S.); diana.chiorean@umfst.ro (D.M.C.); corina.budin@umfst.ro (C.E.B.); ovidiu.cotoi@umfst.ro (O.S.C.); 3 Pathology Department, Clinical County Hospital Mureș, 540136 Targu Mures, Romania; ohii.bianca@yahoo.com; 4 Pneumology Department, Clinical County Hospital Mureș, 540136 Targu Mures, Romania

**Keywords:** melanoma, cancer stem cells, therapy resistance, signaling pathways

## Abstract

Cutaneous malignant melanoma is an extraordinarily aggressive and heterogeneous cancer that contains a small subpopulation of tumor stem cells (CSCs) responsible for tumor initiation, metastasis, and recurrence. Identification and characterization of CSCs in melanoma is challenging due to tumor heterogeneity and the lack of specific markers (CD271, ABCB5, ALDH, Nanog) and the ability of cells to dynamically change their phenotype. Phenotype-maintaining signaling pathways (Wnt/β-catenin, Notch, Hedgehog, HIF-1) promote self-renewal, treatment resistance, and epithelial–mesenchymal transitions. Tumor plasticity reflects the ability of differentiated cells to acquire stem-like traits and phenotypic flexibility under stress conditions. The interaction of CSCs with the tumor microenvironment accelerates disease progression: they induce the formation of cancer-associated fibroblasts (CAFs) and neo-angiogenesis, extracellular matrix remodeling, and recruitment of immunosuppressive cells, facilitating immune evasion. Emerging therapeutic strategies include immunotherapy (immune checkpoint inhibitors), epigenetic inhibitors, and nanotechnologies (targeted nanoparticles) for delivery of chemotherapeutic agents. Understanding the role of CSCs and tumor plasticity paves the way for more effective innovative therapies against melanoma.

## 1. Introduction

Cutaneous melanoma is an extremely aggressive neoplasm, showing increased heterogeneity and a very high potential for metastasis; as a result, the prognosis is very poor in advanced stages. Although many effective therapies for melanoma have emerged in recent decades (including molecularly targeted therapy and immunotherapy), the survival of patients with metastases is still poor [1,2].

An important factor contributing to melanoma’s therapy resistance is its intrinsic heterogeneity. This is due to the very high number of genetic mutations (the highest among all human cancers), generating an exceptional degree of intratumor heterogeneity. Within the same tumor, multiple cell subpopulations with different phenotypes and genotypes can exist, leading to distinct biological behaviors. In addition, the tumor microclimate has a crucial role in the development of melanoma: the signals provided by the stromal, vascular, and immune components at the tumor level can directly influence the growth and survival processes, favoring tumor growth, resistance, and metastasis [3,4].

In this context, the idea of a cell subpopulation with “stem cell” properties emerged, which would be responsible for initiating the tumor process and maintaining it. According to the theory, a very small part of the tumor cells possesses the self-renewal capacity and differentiation. This subpopulation, known as tumor stem cells (CSCs) or melanoma stem cells in this case, is differentiated from the rest of the subpopulations by the expression of some molecular and immunological markers of “stemness” and by the alteration of some signaling pathways [5]. Like the body’s normal stem cells, this subpopulation can divide asymmetrically, giving rise to a diversity of tumor subclones. This tumor plasticity is controlled by epigenetic mechanisms like those in normal adult or embryonic stem cells; alteration of these mechanisms confers the malignant character and phenotype of CSCs [6].

Identification and description of these cells is difficult due to the lack of highly specific markers. Many molecules have been proposed as markers (ALDH1a1, ABCG2, CD44, CD133, CD271, NANOG), but they are also found in non-tumor stem cells and normal melanocytes. As a result, the presence of these markers cannot directly define this subpopulation and rather suggests that the stem-like phenotype is more of a dynamic state that a cell compartment can acquire. In this direction, factors in the tumor microclimate (e.g., hypoxia) that can lead to the acquisition of “stemness” properties have been observed: studies performed on cell cultures have highlighted this property, associating it with improved proliferation and survival. These findings confirm tumor plasticity, which involves tumor cells being able to switch from one state to another, adapting to local conditions to maintain tumor development [7].

The connection between CSCs and the tumor microenvironment provides direct functional adaptability for tumor evolution. Recent studies have identified the fact that only a small subpopulation, predominantly located in the perivascular area, possesses the capacity for self-renewal and tumor growth, while another subpopulation, predominantly located peripherally at the stromal level, possesses the ability to metastasize. These phenotypic properties are not rigid and fixed but are acquired and developed depending on the signals provided by the environment [8].

From a clinical point of view, the presence of these cells with varied properties and increased plasticity may be the explanation for many of the therapeutic failures for melanoma. Stem-like cells have demonstrated a remarkable ability to evade and reject conventional therapies targeted against proliferative components. Thus, proliferation is renewed, and cells can metastasize. However, the identification of signaling pathways and molecular markers offers new therapeutic opportunities [9,10].

Starting from these premises, it is necessary to create an updated synthesis about CSCs in melanoma, with the potential for a more detailed understanding of these cells and to guide new personalized therapeutic strategies. This review aims to present in detail the process of identification and characterization of these cells, the signaling pathways used, their plasticity and origin, the connection with the tumor microclimate, and the functional and therapeutic implications.

## 2. Identification and Characterization of Melanoma Tumor Stem Cells

CSCs are defined as a distinct subpopulation of tumor cells that possess self-renewal and differentiation capabilities, providing tumor diversity and leading to long-term tumor development. While a very small proportion of tumor cells were initially proposed to have such properties, their identification and demonstration has been difficult. Early studies identified “embryonic” properties in some cell subsets within tumors, suggesting the existence of CSCs in melanoma. However, demonstrating these cells has been difficult due to their increased plasticity and the lack of specific markers [11,12].

To identify this subpopulation, studies have used cell surface markers, but also functional tests. A series of molecular markers have been investigated as indicators of stem cells. Among them are membrane glycoproteins such as CD133, the receptor for neurotrophic factors CD271 (p75NGFR), transporters of the ABC class (e.g., ABCG2, associated with the lateral population of cells with Hoechst efflux), adhesion proteins such as variants of CD44, and cytoskeletal proteins of the Nestin type. Aldehyde dehydrogenase (ALDH1 isozyme) activity has also been used as a functional marker of stem cells, based on the Aldefluor assay, given that ALDH-positive cells have been correlated with a stem-like phenotype in many cancers. At the same time, tumorspheres (“melanospheres”) obtained by culturing tumor cells in three-dimensional non-adherent conditions represent an essential in vitro method for the enrichment and testing of stem cell behavior [13,14,15] (Table 1).

The challenges in identifying these cells are represented by the proposed markers are not limited to a restricted cell fraction. For example, a study analyzed the expression of seven markers (ALDH1A1, ABCG2, various variants of CD44, CD133, CD271, and Nestin) in 40 cell cultures of primary strains and 40 tumor fragments, making a comparison with normal melanocytes and fibroblasts. Results showed that most of these markers were widely expressed in melanoma cells in vitro and in situ, and when present, the markers were found in the vast majority of tumor cells, not just a small subset. Also, non-malignant cells, including melanocytes and fibroblasts, were positive for some of these markers, emphasizing the lack of specificity of these cells. These data suggest that the use of these markers alone is insufficient to identify these cells, as tumor cells without stem-like properties and non-tumor cells can express these markers [16]. For example, in studies on the murine melanoma line B16-F10, only ∼0.1% of cells expressed the CD133 marker, and immunomagnetic isolation of this subpopulation revealed superior in vitro proliferative and clonogenic potential compared to CD133-negative cells or the mixed population. In studies with in vivo models in immunocompetent mice, both CD133-positive and CD133-negative cells exhibited dividing properties and tumorigenic capabilities. Such results were also reported for other markers: cells sorted by CD271 showed tumor formation, but CD271-negative populations can form tumors under favorable conditions [17] (Table 1).

In addition to marker expression, CSCs display different functional properties. High cloning potential, the ability to initiate the tumor process in murine models at limited dilution inoculations, and resistance to therapies are the characteristics conferred to CSCs. In melanoma, isolated cells (ALDH-positive or spheroid-forming cells) frequently demonstrated higher tumorigenic capacity compared to cells in the general tumor population. Melanocytic tumor cells exhibiting increased ALDH activity were associated with higher tumor incidence than ALDH-negative cells in both NOD/SCID and gamma null NOD/SCID mouse models. Histologically, some studies have identified melanoma CSCs directly in tumor lesions through the colocalization of pluripotency factors typically characterized by embryonic stem cells [18]. A study have highlighted, in melanoma nodal metastases, the existence of two distinct subpopulations of cells simultaneously expressing OCT4, SOX2, KLF4, and c-MYC markers—one located in the tumor nests and the other in the peritumoral stroma. In some cases, NANOG expression was also detected in these pluripotent cells in the tumor mass. The presence of these multiple CSC subpopulations suggests that even within the same melanoma tumor, stem-like cells with differences in microenvironment and localization may coexist, reflecting an added complexity in their characterization. Furthermore, cell lines obtained from such metastases retained the expression of some stemness factors (e.g., SOX2, KLF4, c-MYC) and were able to form tumorspheres in vitro, confirming the tumor stem cell behavior of these isolated subpopulations [19] (Table 1).

In recent years, melanoma research has led to a paradigm shift from the classical model of static CSCs to the recognition of tumor cell dynamics and plasticity. Evidence that the expression of CSC markers can be induced or regulated by tumor environmental conditions—for example, culture of melanoma cells under hypoxic conditions and acidic pH resulted in increased expression of stem-like markers and improved cell survival—supports the idea that any malignant cell could acquire stem cell traits under certain circumstances. Such findings, along with the observation of generalized marker expression in the tumor population, are congruent with the emerging notion of CSC plasticity and phenotype switching within the cancer cell population. Melanoma shows a remarkable capacity for phenotypic adaptation: single-cell follow-up studies have shown that under the action of a stress (such as treatment with BRAF inhibitors), a part of melanoma cells can non-genetically escape the therapeutic pressure by rapidly entering a persistent state that allows them to proliferate in the presence of the drug. This phenomenon highlights a subpopulation possessing transient traits of increased survival, suggesting that certain properties may be transiently acquired by initially sensitive cells. Overall, different identification methods (multiparametric flow cytometry, single-cell expression analyses, and in vivo models) have contributed to an increasingly detailed profile of melanoma CSCs. Thus, the scientific community agreed that CSCs in melanoma are not static and rare entities but can represent a spectrum of cellular adaptability. As a result, current efforts are focusing on achieving a complex, combined profile of markers and functional elements to differentiate these cells from the non-stem cell population. This integrative approach, combining multiple markers and functional assays, is essential to clarify the identity of CSCs in melanoma and paves the way for their further exploitation for diagnostic and research purposes, independent of signaling pathways or tumor niche analysis [20,21,22].

## 3. Signaling Pathways Involved in Maintaining Stemness in Melanoma

Melanoma contains a subpopulation of tumor cells with stem-like properties (cancer stem cells, CSCs), responsible for self-renewal, plasticity, and resistance to therapies. The molecular mechanisms that maintain this stem-like character involve the reactivation of aberrantly embryonic and adaptive signaling pathways. Thus, it was observed that the hyperactivation of the Wnt, Hedgehog (Hh), and Notch pathways is closely associated with the maintenance of “stemness” properties in melanoma cells. These pro-stemness pathways, together with environmental factors such as tumor hypoxia, create a context favorable to the maintenance of an undifferentiated and aggressive phenotype of malignant cells [23].

**Figure 1:**
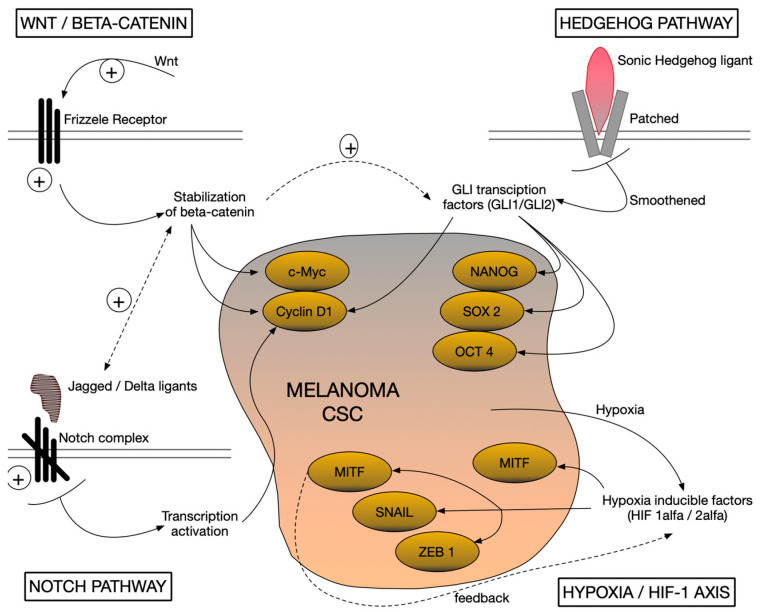
Schematic representation of signaling pathways involved in melanoma cancer stem cells.

***The Wnt/β-catenin pathway*** is a key regulator in stem cell development and maintenance, and its dysregulation contributes to the stemness phenotype in melanoma. Canonical Wnt signaling stabilizes β-catenin, which translocate to the nucleus and activates genes involved in proliferation and survival. In total, 30% of melanomas show abnormal activation of the Wnt/β-catenin pathway, suggesting a specific role for it in maintaining CSCs in this type of cancer. Experimental inhibition of β-catenin (e.g., by restoring suppressor function of APC) reduces the nuclear accumulation of β-catenin and has been associated with decreased ability of tumor stem cells to self-renew, concomitant with a sensitization of them to therapies such as radiotherapy. Frizzled Wnt receptors also play an important role. For example, Frizzled-3 (FZD3) has been shown to be essential for melanoma oncogenesis by non-canonically initiating Wnt-MAPK signaling. Suppression of FZD3 expression reduces melanoma cell proliferation, colony formation, and invasiveness, inhibiting tumor initiation and growth in vivo. These data highlight the Wnt pathway as a mainstay of maintaining the stem-like phenotype in melanoma [24,25] (Figure 1).

***The Notch pathway*** regulates cell differentiation decisions in development and is also involved in the maintenance of melanoma CSCs. The Notch complex (Notch1–4 receptors and Jagged/Delta ligands) is overactivated in melanoma. Notch1, for example, is highly expressed in ~60% of melanomas and nearly absent in normal melanocytes. Continuous activation of Notch signaling supports tumor stem cell self-renewal and survival. Genomic expression studies have correlated the Notch activation signature with poor prognosis in patients with metastatic melanoma. Functional evidence for the role of Notch comes from experiments with γ-secretase inhibitors (which block Notch activation). Specifically, treatment of primary melanoma cells with such an inhibitor (RO4929097) reduced their oncogenic and stem-like properties and suppressed the growth of melanoma tumors in vivo. These observations indicate that Notch signaling maintains the stem-like state of melanoma cells, contributing to tumor aggressiveness [26] (Figure 1 and Table 2).

Notch4 is a membrane receptor that has different roles from the rest of the Notch family: regulation of tumor migration, vascular branching, and differentiation of lymphatic endothelial cells (LECs) during embryonic lymphangiogenesis. Compared to Notch1, Notch4 activation results in a stronger inhibition of LEC migration and alters the expression of a different set of target genes, including factors involved in chemotaxis and suppression of mature lymphatic markers. The activity and influence of Notch4 is carried out predominantly by non-canonical mechanisms, independent of the interaction with the rest of the complexes. These features support the involvement of Notch4 in the maintenance of an immature, migratory, and resistant cell phenotype to differentiation signals. Thus, Notch4 is a relevant candidate for investigating the molecular mechanisms underlying the plasticity and invasiveness of malignant stem cells [27] (Figure 1 and Table 2).

***The Hedgehog (SHH-GLI) pathway***, known for its essential role in embryogenesis and adult tissue homeostasis, is also involved in the maintenance of tumor stem cells in melanoma. Aberrant activation of the Hedgehog cascade (Sonic Hedgehog ligand—Patched/Smoothened receptors—GLI transcription factors) promotes CSC self-renewal, initiates tumorigenesis and promotes melanoma progression and metastasis. SHH-GLI signaling directly stimulates melanoma cell proliferation and survival, and blocking this pathway has an inhibitory effect on the stemness phenotype. For example, experimental inhibition of Hh-GLI signaling led to decreased spheroid self-forming capacity and tumorigenicity of the ALDH^high^ (CSC-enriched) cell subpopulation. Furthermore, targeting the transcription factors GLI1/GLI2 (nuclear effectors of the Hh pathway) was able to reverse the treatment resistance of melanoma cells refractory to BRAF inhibitors and even prevent the acquisition of resistance to chemotherapy. This suggests that, in addition to maintaining stemness, the Hedgehog pathway also confers survival advantages under therapeutic stress, underscoring its dual importance in melanoma biology [28,29] (Figure 1 and Table 2).

***Tumor hypoxia and the HIF-1 axis*** represent another critical mechanism supporting the stem-like phenotype in melanoma. The hypoxic microenvironment within the tumor stabilizes hypoxia-inducible factors (HIF-1α/2α), which act as key transcription factors in the adaptation of cells to low oxygen. HIF-1α orchestrates hypoxic responses by remodeling gene expression. On the one hand, it induces pro-angiogenic genes and changes metabolism toward glycolysis, facilitating tumor cell survival; on the other hand, it directly influences the maintenance of their undifferentiated character. In melanoma, HIF-1α can stimulate the expression of the melanocyte-specific transcription factor MITF (microphthalmia-associated transcription factor) even in hypoxia, which paradoxically favors tumor growth. In addition, hypoxia-active HIFs trigger the epithelial–mesenchymal transition (EMT) programs by upregulating factors such as TWIST, SNAIL, and ZEB1, conferring a more migratory and invasive status to cells, traits associated with CSCs. The importance of the hypoxia–HIF axis in the maintenance of stemness is also supported by functional experiments. It has been shown that the reduction of HIF-1α expression in melanoma cells (for example by genetic silencing) leads to the decrease in the expression of hypoxic target genes and to the diminution of the stem-like characteristics of CSCs. Altogether, hypoxia-mediated signaling contributes to a survival and plasticity phenotype, allowing tumor cells to persist in hostile niches and regenerate the tumor [30] (Figure 1 and Table 2).

Last but not least, ***pro-inflammatory paracrine signals*** from the tumor microenvironment can activate cellular pathways favoring the maintenance of CSCs in melanoma. For example, cytokines released by infiltrating immune cells (such as neutrophils or tumor-associated macrophages) can trigger cascades such as JAK/STAT3 and NF-κB in tumor cells, enhancing their survival and stemness program. A chronic inflammatory tumor microenvironment, rich in factors such as IL-6, has been reported to activate STAT3 and concomitantly stimulate the Wnt pathway in melanoma, creating a vicious cycle that promotes CSC self-sufficiency and tumor progression [31]. Likewise, lipid mediators in the TME can directly influence stem-like cells. Bioactive sphingolipids, such as sphingosine-1-phosphate (S1P), have been shown to activate pro-cancer signaling through Stat3 and Akt, increasing the expression of anti-apoptotic proteins (Bcl-2, Bcl-xL) and protecting tumor stem cells from cell death. In melanoma, S1P signaling confers the increased resistance of CSCs to apoptosis and facilitates the interactions of these cells with the stroma, contributing to their persistence in the tumor. Thus, microenvironmental factors may cooperate with tumor cell intrinsic pathways to reinforce the stem-like state and fuel tumor heterogeneity [32].

Thus, the described aberrant signaling pathways converge toward the maintenance of a fraction of melanoma cells with stem-like characteristics. These cells possess an increased capacity for tumor initiation, adaptation, and resistance, contributing to melanoma aggressiveness and post-therapeutic relapses. A deep understanding of how each signaling pathway participates in the maintenance of “stemness” in melanoma—and the interactions between them—is essential for elucidating the mechanisms of tumor progression and may guide the future development of strategies to combat these malignant pluripotent cells. The scientific community is making strides toward decoding CSC vulnerabilities in melanoma, without addressing specific therapeutic interventions in this section. Analyzing the molecular aspects of “stemness”, it is clear that the resistance of tumor stem cells in melanoma is related to the reactivation of some signaling pathways [33].

## 4. Tumor Plasticity and Cellular Origin in Melanoma

Melanoma is characterized by remarkable phenotypic heterogeneity and exceptional cellular plasticity. Tumor cells can exist in a wide spectrum of differential states and can dynamically transform between distinct phenotypes. Regardless of genomic classification, melanoma can switch from a proliferative and differentiated phenotype (marked by high MITF expression) to an invasive and dedifferentiated phenotype (characterized by low MITF and neural crest stem cell markers such as NGFR). In fact, at least six distinct cellular states have been identified in melanoma, ranging from hyperdifferentiated/pigmented and melanocytic cells (MITF^high^/AXL^low^), passing through transient intermediate states (including a CD36^+^ “starved” phenotype) to fully dedifferentiated states such as neural crest stem cell-like subpopulations (MITF^low^/NGFR^high^). This phenomenal plasticity allows malignant melanocytic cells to adopt transdifferentiation characteristics, for example toward vascular or neuronal types, reflecting the reactivation of genetic programs specific to the embryonic neural crest. Consistently, melanoma progression has been associated with a gradual dedifferentiation of tumor cells and blockage of gene regulatory networks. This behavior indicates that tumor stem cell status in melanoma is not a static property of a fixed subpopulation, but rather a dynamic phenotype that cells can acquire or lose. The cellular origin of melanoma is closely related to its plasticity. Normal melanocytes originate from multipotent neural crest cells. In adult tissues, melanocyte homeostasis is maintained by resident melanocyte stem cells. Studies in murine models have demonstrated that melanoma can arise either from such melanocyte stem cells or directly from differentiated mature melanocytes, depending on the tissue context and the combination of oncogenic mutations or suppressor gene inactivation involved [34,35,36].

This cell variability suggests that clinicopathological subtypes of melanoma may exhibit different starting points. Consequently, different cell subpopulations can coexist in terms of degree of differentiation, some with proliferative characteristics, others with an invasive and stem-like profile. A hierarchical model was initially proposed, in which only a small subset of tumor cells possess tumor initiation and maintenance capacity. Experimental evidence contradicts this strictly hierarchical view: virtually any melanoma cell can have tumor-initiating potential and reconstitute tumor phenotypic heterogeneity if placed under the appropriate conditions. Thus, melanoma appears to operate based on a non-hierarchical plastic model, in which stemness in the tumor is flexibly distributed and acquired, rather than confined to a stable clone. However, the existence of different functional states indicates a pseudo-hierarchical organization. Only certain cell subpopulations participate in the growth of the primary tumor, and others are predominantly responsible for the initiation of metastases [37,38].

Overall, tumor plasticity and cellular origin in melanoma are two interconnected aspects: inheritance from the neural crest confers on melanocytes (and melanoma) an intrinsic propensity to change cellular identity, allowing the tumor to evolve through a diversity of phenotypic states and adapt its aggressiveness during malignant progression.

## 5. Interaction with the Tumor Microenvironment (TME)

Melanoma tumor stem cells (CSCs) do not exist in isolation but evolve in a complex tumor microenvironment (TME), made up of stromal cells (such as tumor-associated fibroblasts—CAFs), infiltrated immune cells, the vascular network, the extracellular matrix, and numerous soluble factors (cytokines, chemokines, growth factors). Communication between CSCs and these components of the tumor niche plays a very important role in melanoma development. Thus, CSCs actively shape the surrounding microenvironment by removing molecular factors, respond to signals from stromal cells, creating a dynamic dialogue between the tumor stem population and the TME [39,40].

***Tumor stromal activated fibroblasts (CAFs)*** are partners of CSCs, creating a favorable environment for tumor growth and invasion. In melanoma, a close interaction between these has been observed, with recruitment of normal dermal fibroblasts by malignant cells and their reprogramming to a pro-tumor CAF phenotype. Signaling through the protein CCN2 (also known as CTGF) released by tumor cells can activate surrounding fibroblasts, inducing expression of the stemness marker Sox2 in them and turning them into activated CAFs. These CAFs in turn secrete pro-tumor molecules (growth factors, cytokines, and microRNAs), which participate in the aggressiveness of melanoma [41]. A relevant example is microRNA miR-214, found in elevated levels in melanoma CAFs; conditioned medium or extracellular vesicles (exosomes) derived from miR-214-rich CAFs have been shown to stimulate melanoma cell migration and invasion. Tumor cells can in turn induce overproduction of miR-214 in CAFs, which is then released and taken up by malignant cells. In addition to promoting invasion, CAFs directly contribute to maintaining the stemness properties of CSCs. It has been reported that subpopulations of CAFs expressing the CD44 marker can support self-renewal of melanoma CSCs through direct cell–cell contacts, which simultaneously increases the tumor’s resistance to therapies. Furthermore, signaling from CAFs can modulate the plasticity of CSCs, e.g., the pathway Notch1 from tumor-associated fibroblasts acts as a stem phenotype switch. In addition, overexpression of Notch1 in CAFs inhibits the stemness of tumor cells, while inhibition of Notch1 in CAFs leads to increased expression of stemness markers (Sox2, Oct4, Nanog) in melanoma CSCs. Altogether, the close pathological interaction between melanoma CSCs and CAFs in the tumor stroma creates a mutually beneficial signaling circuit for the tumor that favors malignant progression [42] (Table 3).

Melanoma CSCs profoundly influence the composition and function of the ***tumor immune infiltrate***, remodeling it in a pro-tumor sense. CSCs recruit and educate immune cells in situ toward tolerogenic or tumor-promoting phenotypes [43,44].

Tumor-associated macrophages (TAMs) can be attracted to the lesion by chemokines and interleukins secreted by CSCs, which are then polarized toward the M2 (immunosuppressive) state under the influence of these factors. In turn, M2 macrophages release anti-inflammatory cytokines (TGF-β, IL-10, etc.) and enzymes that remodel the matrix, creating a protective microenvironment that supports the survival of CSCs and the maintenance of their stemness [45]. This is a reciprocal exchange—CSCs condition macrophages to a pro-tumor status. Similar mechanisms involve other suppressor myeloid cells. Immature myeloid cells (MDSCs) are attracted by CSCs into the tumor, where they exert their immunosuppressive function and contribute to the maintenance of melanoma stemness by secreting factors such as IL-6 that activate the STAT3 pathway in tumor cells. Thus, a vicious circle is created: CSCs stimulate the recruitment of immunosuppressive populations (such as MDSCs, M2 macrophages, and regulatory T lymphocytes), which in turn increase the stem phenotype and aggressiveness of melanoma [46] (Table 3).

CSCs have been shown experimentally to secrete a cocktail of soluble factors (TGF-β, IL-6, IL-8) that attract neutrophils from the circulation and reprogram them to a pro-tumor N2 phenotype. Thus, neutrophils undergo functional changes. The increase the expression of activation markers (CD11b, CD66b) and survival signals (STAT3/ERK pathway is activated), produce large amounts of reactive oxygen species (ROS), and secrete metalloproteinases (MMP-9) and inflammatory cytokines. A feed-forward effect was observed: CSC-conditioned neutrophils can reinforce the stemness of melanoma cells, increasing their ability to form tumor spheres and the level of resistance markers (e.g., ABCG2 pump). Therefore, melanoma CSCs actively remodel the tumor immune landscape, subverting infiltrating immune cells to states that support tumor growth and thereby maintaining a permissive niche in the TME [47] (Table 3).

CSCs also interact dynamically with the physical infrastructure of the tumor—the extracellular matrix (ECM) and the microvascular network. The tumor ECM is not just a simple support but is reorganized by cancer stem cells. CSC-rich melanoma frequently exhibits elevated levels of proteolytic enzymes (MMP-2 and MMP-9), which destroy the matrix component. By digesting collagen and other components of the ECM, CSCs make their way through the host tissue and create spaces for the process of angiogenesis. In addition, these cells use matrix adhesion receptors (integrins and proteoglycans) to perceive the composition and stiffness of the microenvironment and to adapt their behavior [48,49,50,51].

Vascularization is essential for the supply of oxygen and nutrients to the growing tumor mass, and melanoma CSCs are shown to be orchestrators of angiogenesis. They produce significant amounts of pro-angiogenic factors (VEGF, angiopoietins, and Tie2) that act on nearby endothelial cells, stimulating their proliferation and migration to form new capillaries. In preclinical studies, CSC subpopulations (ABCG2^+^, CD133^+^, etc.) were highlighted by the intense secretion of such factors and by the ability to form vascular tubes in vitro. More than that, these subpopulations are involved in a phenomenon called vascular mimicry (VM), through which CSCs can partially transdifferentiate into an endothelial-like phenotype, organizing into tubular structures that perfuse the tumor independently of host vessels. The VM phenomenon, along with classic angiogenesis, expands the network of channels through which the tumor feeds and metastasizes. Thus, the CSCs working with the surrounding vascular system. They emit signals that give birth to new vessels or alternative vascular structures, and the formed vessels and endothelial cells emit pro-tumor feedback that intensifies the stemness features of malignant cells [52,53,54,55].

Soluble molecules in the TME mediate a large proportion of CSC–environment interactions. Cytokines and growth factors secreted by CSCs shape the behavior of fibroblasts and surrounding immune cells (TGF-β, IL-6, IL-8), while signals emitted by the stroma (CAF-derived factors or immune cells) can maintain or even pronounce the melanoma stem phenotype. A microenvironment rich in TGF-β is favorable to both reactive fibroblastogenesis and immune evasion, and CSCs take advantage of this molecule. Under tumor hypoxia, TAM macrophages and regulatory T lymphocytes release large amounts of TGF-β that stimulate melanoma cells to increase GCS enzyme expression, which is associated with the expansion of the CSC population. In addition to cytokines, extracellular vesicles (exosomes) secreted by CSCs represent an important mode of intercellular communication in the TME. Exosomes produced by CSCs contain microRNAs and proteins that can reprogram responsive cells. For example, microvesicles derived from melanoma CSCs can transfer microRNA miR-592 to non-stem tumor cells, triggering activation of the MAPK/ERK pathway in them and enhancing their metastatic potential [56,57,58,59] (Table 3).

Last but not least, the local physical and chemical parameters—hypoxia and acidity—also influence the interactions of CSC with the environment. Hypoxic areas within the melanoma contain increased numbers of CSCs. Hypoxia activates transcription factors (HIF-1α) that alter the metabolism of these cells and induce the secretion of proangiogenic and immunomodulatory factors. A low extracellular pH can also stimulate CSCs and the release of different exosomes, loaded with signals favoring survival in an acidic environment. These stress conditions in the TME therefore act as selective forces that can intensify the cooperation between CSCs and the stromal component, orienting the microenvironment toward supporting tumor progression [60,61,62].

The melanoma tumor microenvironment creates an ecosystem in which CSCs are the command center that remodels the tumor niche, while being influenced by surrounding cells and factors. Complex interactions with CAFs, immune cells, ECM, and the vasculature create a vicious cycle fueling melanoma aggressiveness and heterogeneity. Through these reciprocal connections, CSCs not only benefit from a favorable (nutritional and immunological) refuge for self-renewal and survival but educate the stromal component to work to the advantage of the tumor. A deep understanding of this CSC–TME molecular dialogue provides mechanistic insight into why melanoma is so refractory and metastatic, independent of the simple biology of isolated malignant cells [63,64,65,66].

## 6. Therapeutic Implications and Targeting Strategies of CSCs

Malignant melanoma is characterized by a pronounced heterogeneity and resistance to treatments, to which a rare subpopulation of cells—tumor stem cells (CSCs)—make a major contribution. The presence of CSCs in melanoma correlates with poor prognosis and frequent therapeutic failures. Different biomarkers (CD133, CD271, and ALDH activity) identify aggressive melanoma subpopulations associated with stem phenotype, although no marker is exclusive to these cells, making their precise isolation difficult. However, these stem-like cells employ survival mechanisms such as immune evasion and vasculogenic mimicry, allowing melanoma to survive in hostile conditions. Evading immune surveillance, CSCs can persist even under modern immune therapies, constituting a reservoir for relapse and distant metastasis [67,68,69,70].

**Figure 2:**
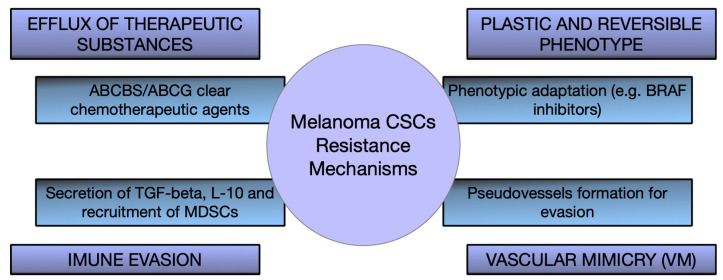
Therapeutic resistance mechanisms of melanoma CSCs.

CSCs are a major factor in therapy resistance and tumor recurrence in melanoma. Incomplete elimination of these cells during treatments (chemotherapy, targeted therapies, or immunotherapy) leads to therapeutic failure and disease progression [71,72]. Preclinical studies confirm that only subpopulations of cells bearing stem markers (CD133+ or CD20+) can regenerate tumors in mice and show increased resistance, highlighting the role of CSCs in tumor maintenance. These results explain why patients with metastatic melanoma achieve initial remissions followed by relapses: residual CSCs repopulate the tumor with new, often more aggressive cells, making their presence a negative prognostic indicator and a major obstacle to achieving durable clinical responses [73,74] (Figure 2).

Melanoma stem cells maintain their properties through aberrant activation of embryonic and survival signaling pathways (Wnt/β-catenin, Hedgehog, and Notch). Blocking these essential pathways has shown promising effects in preclinical models. Inhibition of Notch signaling with a γ-secretase inhibitor reduces the stem-like phenotype and tumorigenic capacity, and blocking the Hedgehog pathway (e.g., with Vismodegib) induces CSC death and slows tumor growth. These strategies are still experimental but illustrate the possibility of making CSCs vulnerable by disrupting the signaling programs that maintain them. Since these pathways also intervene in normal stem cell functions, they must be targeted as selectively as possible (e.g., through targeted delivery systems) to minimize toxic effects on healthy tissues [75,76,77,78,79] (Figure 2).

Modern immunotherapy has dramatically improved melanoma prognosis, but the persistence of CSCs with an immune-evasive phenotype may limit complete response. Combination strategies to simultaneously target CSCs and potentiate antitumor immunity are being investigated. A key direction is the development of vaccines that train the immune system against CSC-specific antigens (e.g., ALDH or stemness factors such as Sox2 and Nanog). In preclinical studies, such an anti-CSC vaccine combined with checkpoint blockade (anti-PD-1 and anti-CTLA-4) generated a strong immune response and eradicated CSC populations, causing tumor regression and preventing relapses, without affecting normal stem cells [80,81,82,83] (Figure 2).

Dysregulation of epigenetic networks contributes to the maintenance of the “stemness” state of melanoma cells, so epigenetic modulators can force the differentiation of CSCs or make them more vulnerable to treatments. Inhibitors of histone deacetylases (HDACs) and DNA methyltransferases, alone or in combination with other therapies, have demonstrated preclinical potential to increase tumor immunogenicity and counteract acquired resistance to treatment. A notable example is the reprofiling of disulfiram (an anti-alcoholism drug). In the presence of copper ions, this agent inhibits ALDH (a functional marker of CSC) and the proteasome, causing the selective death of subpopulations of stem-like cells. In preclinical models, the disulfiram–copper combination eradicated the CSC pool, preventing relapse and metastasis and resensitizing tumors refractory to chemo- and radiotherapy [84,85] (Figure 2).

A major challenge in eliminating CSCs is the efficient and selective delivery of drugs to these cells. Nanotechnology offers promising solutions to directly target CSCs. For example, nanoparticles functionalized with specific ligands (antibodies or peptides) can recognize the surface markers of CSCs and can locally deliver lethal doses of chemotherapeutic agents or interfering RNA, with minimal impact on normal cells. Loading a Hedgehog pathway inhibitor into CSC-targeted nanoparticles blocks stemness signaling directly at the source, avoiding systemic exposure to drug toxicity. In addition, nanovectors can cross physiological barriers (such as dense tumor stroma) and controllably release active substances into the tumor microenvironment, where CSCs often hide in hypoxic niches. Although these approaches are still experimental, they offer the prospect of combining cytotoxic attack on the tumor mass with targeted eradication of the CSC reservoir in order to improve the efficacy of melanoma treatment [86,87,88].

## 7. Conclusions

Tumor stem cells (CSCs) in melanoma are a rare subpopulation of cells with a major role in its development. Through their phenotypic plasticity and dynamic interaction with the environment, they achieve intratumoral heterogeneity and increased aggressiveness. These cells possess a remarkable capacity for self-renewal and differentiation, resistance to therapy, and transition to metastasis.

Attacking these tumor stem cells in melanoma represents the direction of therapy development. Therapeutic options include blocking aberrant signaling pathways responsible for the stem cell phenotype, creating immunotherapies against specific antigens or blocking immune checkpoints.

Current knowledge emphasizes the need to study these cells in much more detail to identify specific markers that allow targeting these cells without affecting normal tissue. It is also essential to implement combined approaches (destruction of tumor stem cells but also of the tumor bed) to reduce the number of relapses and metastases.

Consolidating and improving current knowledge could be the key to identifying new therapies capable of overcoming tumor resistance and significantly improving patient prognosis.

## Figures and Tables

**Table 1:** Markers for identifying tumor stem cells (CSCs) in melanoma.

MARKER	TYPE	PROPOSED ROLE	SPECIFICITY AND OBSERVATIONS
**CD133 (PROMININ-1)**	Membrane marker	Self-renewal and tumorigenesis	Limited to a small fraction of cells, but also present in non-stem cells
**CD271** **(P75 NGFR)**	Membrane marker	Associated with invasion and survival	Present in CSCs, but also in other malignant cell populations and normal melanocytes
**ABCB5/** **ABCG2**	Functional marker	Therapeutic resistance through toxic efflux	Expressed in CSCs and associated with resistance, but not specific to the CSC subpopulation
**ALDH1**	Enzymatic marker (high metabolic activity)	Self-renewal and therapeutic resistance	Associated with the CSC phenotype, but also detected in non-CSC cell populations in melanoma
**OCT4, SOX2, NANOG**	Transcription factor	Maintenance of pluripotency	Variable expression in CSCs and in tumor cells with increased plasticity

**Table 2:** Comparative table of molecular pathways involved in the maintenance of melanoma CSCs.

MOLECULAR PATHWAY	KEY COMPONENTS	EFFECTS ON CSCS	THERAPEUTIC IMPLICATIONS
**WNT/β-CATENIN**	Nuclear β-catenin, FZD3	Self-renewal, survival specific	β-catenin inhibitors
**NOTCH**	Notch receptors, Jagged/Delta ligands	Maintenance of undifferentiation and self-renewal	γ-secretase inhibitors
**HEDGEHOG (HH)**	SHH-GLI	Proliferation, invasion, therapeutic resistance	Vismodegib-like inhibitors
**HIPOXIA/HIF-1**	HIF-1α, TWIST, SNAIL, ZEB1	EMT, survival, and metabolic adaptation	Agents that block HIF activity

**Table 3:** Comparative table on the role of the tumor microenvironment in CSC-mediated melanoma progression.

MICROENVIRONMENT COMPONENT	SECRETED FACTORS	ROLE IN INTERACTION WITH CSC	POTENTIAL THERAPEUTIC TARGET
**CAF SITES**	miR-214, pro-tumor cytokines	Support of CSC invasion and self-renewal	Inhibitors of CAF activation
**M2 MACROPHAGES (TAM)**	TGF-β, IL-10	Immunosuppression and protection of CSCs	TAM repolarization toward the M1 phenotype
**MYELOID SUPPRESSOR CELLS (MDSCS)**	IL-6	CSC maintenance via STAT3 activation	IL-6/JAK-STAT inhibitors
**NEUTROPHILS N2**	ROS, MMP-9	Promotion of invasion and potentiation of CSCs	Blocking chemotactic factors (IL-8)

## Data Availability

Not applicable.
